# Impact of a Fruit–Vegetable Preparation Fortified with Potato Starch Resistant Dextrin on Selected Health Indicators in Overweight Children

**DOI:** 10.3390/nu16142321

**Published:** 2024-07-18

**Authors:** Katarzyna Śliżewska, Michał Włodarczyk, Renata Barczyńska, Janusz Kapuśniak, Piotr Socha, Aldona Wierzbicka-Rucińska, Aneta Kotowska

**Affiliations:** 1 Institute of Fermentation Technology and Microbiology, Department of Biotechnology and Food Sciences, Technical University of Lodz, Wolczanska 171/173, 90-924 Lodz, Poland; mwlodarczyk94@outlook.com; 2 Department of Dietetics and Food Studies, Faculty of Science and Technology, Jan Dlugosz University, Armi Krajowej 13/15, 42-200 Czestochowa, Poland; r.barczynska-felusiak@ujd.edu.pl (R.B.); j.kapusniak@ujd.edu.pl (J.K.); 3 The Children’s Memorial Health Institute, Aleja Dzieci Polskich 20, 04-736 Warsaw, Poland; p.socha@ipczd.pl (P.S.); a.wierzbicka-rucinska@ipczd.pl (A.W.-R.); a.kotowska@ipczd.pl (A.K.)

**Keywords:** bacterial enzymes, SCFA, BCFA, gut microbiota, obesity

## Abstract

Developing modified dietary fibers that maintain prebiotic benefits without significantly affecting meal taste is of high importance in the midst of the obesity pandemic. These benefits include regulating the composition of gut microbiota, increasing feelings of fullness, and improving human metabolic parameters. This study investigated the use of a resistant dextrin (RD) derived from potato starch, which possesses prebiotic properties, as a potential additive in vegetable–fruit preparations that aid weight loss and improve health markers in overweight children. HPLC was employed to examine metabolites like lactic acid, short-chain fatty acids (SCFAs; formic, acetic, propionic, butyric, and valeric acids), and branched-chain fatty acids (BCFAs; isobutyric and isovaleric acids). The activities of α-glucosidase, β-glucosidase, α-galactosidase, β-galactosidase, and β-glucuronidase enzymes in fecal samples were measured using spectrophotometric analysis at a wavelength of 400 nm. Incorporating the RD into vegetable–fruit preparations yielded favorable outcomes in terms of increased concentrations of the tested metabolites (SCFAs and BCFAs) and enhanced fecal enzyme activities after 6 months of consuming the preparations. Furthermore, these effects were found to last for an extended period of 3 months even after discontinuing the treatment. The study has shown that including RD into vegetable–fruit preparations enhances the metabolic parameters of obese and overweight children, hence providing a strong rationale for the widespread usage of these preparations in the industry.

## 1. Introduction

In modern countries, people began to pay more attention to maintaining healthy dietary habits of society as the increased rate of childhood obesity was observed. The impact of adolescent obesity on the body is profound and multifaceted, affecting nearly every system. It might cause the early onset of type 2 diabetes, cardiovascular diseases, and metabolic syndrome, as well as psychological issues such as depression and low self-esteem [1,2,3]. Moreover, the risk of these conditions extends into adulthood, significantly increasing the likelihood of lifelong health problems and reduced life expectancy [4]. There are various types of foods dedicated to children, but there is still a lack of functional foods that boost their overall health, e.g., by enhancing gut microbiota and maintaining a healthy weight. Several types of fiber have an unfavorable influence on the sensory properties of meals or the digestive system, which is why the production of modified dietary fibers, which have little or no effect on the taste of meals and have all the advantages of prebiotics, such as modulating gut microbiota composition, promoting satiety, and enhancing human metabolic parameters, is of high importance.

The World Health Organization and the National Center for Chronic Disease Prevention and Control revealed a significant surge in the occurrence of metabolic diseases in high-income countries, demonstrating that obesity has emerged as a worldwide pandemic in the twenty-first century [5,6]. According to the newest data, in 2022, more than 2.5 billion individuals were overweight, with 16% being obese (defined as a body mass index (BMI) of 30 or above) [7,8,9]. This is relevant because overweight children have a significantly higher likelihood of developing obesity in adulthood compared to non-overweight children, which will directly impact the number of people experiencing health problems associated with obesity [8,10]. Several predictions state that the frequency of juvenile obesity might double by 2030 and continue to climb beyond that level [6,11,12].

Lifestyle changes such as a preference for a sedentary lifestyle or less physically demanding work, high levels of stress, lack of time to prepare nutritious meals, and other similar circumstances contribute to the onset of metabolic diseases [3]. Several studies, such as those presenting a comparison of the gut microbiota of normal-weight and obese people or examining the effects of various foods on the microorganisms in the digestive tract, have confirmed the long-held belief that one’s diet affects the composition of one’s gastrointestinal microbiota [13,14,15,16,17]. In addition to aiding in energy metabolism, gut microorganisms produce a wide range of bioactive compounds, including lactic acid, SCFAs, and BCFAs, which all play a role in multiple metabolic pathways and have been shown to have anti-inflammatory, anticarcinogenic, and antioxidative effects (*Lactobacillus*, *Bifidobacterium*, and others) [18,19,20]. On the other hand, a poor diversity of gut microbes favoring particular genera (*Bacteroides*, *Clostridium*, *Escherichia*, *and Enterococcus*) may lead to decreased beneficial molecule production and the increased synthesis of potentially hazardous substances [21,22] such as bacterial enzymes, which are mostly reductases and hydrolases and are often associated with the creation of carcinogens and other hazardous chemicals. The highest activities are exhibited by β-glucuronidase (EC 3.2.1.31), β-glucosidase (EC 3.2.1.21), and β-galactosidase (EC 3.2.1.23) [23].

The host benefits from SCFA production because of their ability to regulate energy metabolism, the immune system, and the intestinal barrier [3,24]. Nevertheless, BCFAs are linked to protein fermentation, which may raise the risk of colon cancer due to the buildup of potentially hazardous bioactive chemicals [25]. Furthermore, to aid in digestion, bacteria secrete a wide range of enzymes, (i.e., reductases and hydrolases), which can contribute to the production of beneficial SCFAs, but they may also be responsible for the synthesis of hazardous or carcinogenic substances [23,26]. Epidemiological studies suggest that the enhanced activity of β-glucuronidase and β-glucosidase is commonly linked to alterations within the makeup of the intestinal microflora, where a compositional shift occurs, in which less desirable species become more prevalent in the environment [27]. The aforementioned bioactive compounds have such a significant effect on the human body that they may be used as health markers.

In light of this information, this research set out to investigate the effects of fruit–vegetable preparations enriched with a fiber preparation from potato starch on bacterial enzymes and the composition of fatty acids in fecal samples of a cohort of overweight children without health issues.

As a result, the concentrations of SCFAs were assessed using high-performance liquid chromatography (HPLC), including lactic acid and SCFAs (formic, acetic, propionic, butyric, and valeric acid). Aside from that, the levels of BCFAs were evaluated. Moreover, the activities of selected fecal enzymes were determined. Statistical analysis was also used on the data gathered to confirm if any connections between the various health markers discussed could be found.

## 2. Materials and Methods

The RD used in the study was created at the Jan Dlugosz University in Czestochowa. It was derived from starch, distinguished by the fact that it is the result of the simultaneous dextrinization and crosslinking of potato starch in the presence of citric acid used in the amount of up to 0.1% of dry weight of starch and inorganic acid, preferably hydrochloric acid, at a temperature of 110–150 °C for up to 4 h, characterized by a solubility of 63%, pH of 5, with a content of reducing sugars of 17%, average DP of 25, average chain length of 10.5, and the content of resistant fractions in the preparation determined by the Englyst method was 60%. Patent number: 220965.3.1.

### 2.1. Biological Material

The fecal samples were collected from 100 participants (47 boys and 53 girls) of the PreSTFibre4kids research program (overweight and obese children, patients of The Children’s Memorial Health Institute in Warsaw) and from 11 healthy children with normal weight (control group) aged 6–10 years (Table 1). Overweight criteria that were used included BMI z-score between 85th and 97th percentiles, >1 SD according to the WHO, while obesity criteria were BMI z-score > 97th percentile and >2 SD according to WHO.

Children were voluntarily recruited from October 2021 to January 2022. At the beginning of the study, participants received a physical examination, anthropometric measurements (weight, height, and waist), and additional medical tests (fasting glucose, insulin, total cholesterol, triglycerides, apolipoprotein, uric acid, alanine, and aspartate aminotransferases). Bioimpedance measured the body fat of the children. Anthropometric measurements were reassessed at the completion of the following intervention periods (24 weeks) and 12 weeks afterward. Patients from PreSTFibre4kids were recruited from across Poland; however, the control group consisted of volunteers only from central Poland (mainly Lodz voivodship).

All parents and children received dietary guidance from the dietician, which recommended a normoenergetic diet and daily low-level physical exercise for at least 1 h. Next, individuals were randomly assigned to two groups. For 24 weeks, children in the RD group consumed Tymbark fruit and vegetable preparations (Tymbark-MWS, Tymbark, Poland) with 10 g of RD from potato starch, while those in the control group were given the same preparations without RD. Participants received identical foil packets of preparations and consumed two packets per day in both groups. The mousse composition and RD replenishment methods are detailed in Table 2. The process of randomization was carried out by an investigator independently, without any involvement from the participants. Additionally, both the participants and the study staff were uninformed of the specific treatments being administered.

During the research period, 17 participants resigned; thus, the total number of participants who finished the study was 83.

Procedures adhered to institutional and national research committee ethical requirements per the Helsinki Declaration and its revisions or comparable criteria. The Children’s Memorial Health Institute in Warsaw, Poland’s Ethics Committee accepted the study (18/KBE/2021, 28 April 2021). Participants and their parents received a detailed overview and gave written consent before registration.

The study included children between the ages of 6 and 10, of both genders, who were classified as overweight or obese based on the criteria provided by the World Health Organization. Additionally, the children were required to have obtained written agreement from their parents. The exclusion criteria encompassed allergies to mousse components, malabsorption syndrome, organ failure, food neophobia, and other health conditions that could potentially hinder the study procedures or pose a risk to safety. Prior to and during the collection of fecal samples, none of the subjects had taken antibiotics or probiotics during a period of six months. The stool specimen collection package included a Styrofoam box with a sterile tube, spatula, and ice pack for feces collection. Samples were transported to the lab at −20 °C and stored at −80 °C until use.

The study was divided into the 3 following phases:

Stage 1: Fecal samples from participants of the study were collected before any dietary intervention.

Stage 2: Intervention consisted of administration of the vegetable–fruit preparation enriched with the RD to study participants for 6 months. The study was double blind and randomized. A total of 42 children received vegetable–fruit preparation enriched with the RD (10 g/day), while 41 children were given vegetable–fruit preparation enriched with glucose as a control. Participants were advised to consume 2 packages of the preparation (100 g each, 5 g of RD in each), one in the morning and one in the evening. Participants were able to choose 1 of 3 flavors of the preparation:

apple, carrot, and quince

apple, cherry, carrot, and banana

apple, peach, and parsnip

Information about the composition of the preparations was gathered in Table 2.

Fecal samples were gathered after 6 months of consistent administration of the preparation.

Stage 3: Fecal samples were collected again after 3 months after the last administration of the vegetable–fruit preparation enriched with the RD to investigate whether the effects were long-lasting.

### 2.2. Activity of Fecal Enzymes

Initial steps included vortexing a suspension of 0.7 g of sample in 0.2 M phosphate buffer at a volume of 3.5 mL (Vortex RS-VA 10, Phoenix Instrument, Garbsen, Germany). The samples were then sonicated (time = 2 min; amplitude = 60; pulse = 6 s; Cole-Parmer Instrument Co., Vernon Hills, IL, USA). After centrifuging the samples (12,000 rpm; time = 20 min; Centrifuge MPW-251, MPW, Warsaw, Poland), the supernatant was placed in clean Eppendorf tubes.

Spectrophotometric techniques were used to assess the fecal enzyme activity of α-glucosidase, β-glucosidase, α-galactosidase, β-galactosidase, and β-glucuronidase. The study’s protocols relied on the reaction of α-glucosidase, β-glucosidase, α-galactosidase, β-galactosidase, and β-glucuronidase with 4-nitrophenyl α-D-glucopyranoside (TCI, Tokyo, Japan), 4-nitrophenyl β-D-glucopyranoside (TCI, Tokyo, Japan), 4-nitrophenyl α-D-galactopyranoside (TCI, Tokyo, Japan), 4-nitrophenyl β-D-galactopyranoside (TCI, Tokyo, Japan), and 4-nitrophenyl β-D-glucuronide (TCI, Tokyo, Japan), respectively.

The enzymes contained in the feces were used with substrates designed specifically for them. About 0.25 milliliters of sample, 0.05 milliliters of substrate solution (20 mL of concentration), and 0.5 milliliters of phosphate buffer (pH = 7, 0.02 M) made up the reaction mixture. Various enzymes were incubated at 37 °C for either 15 min (α-glucosidase, α-galactosidase, and α-glucuronidase) or 60 min (β-glucosidase and β-galactosidase). The sample color shift to yellow verified the effect. The color intensity was related to the quantity of liberated p-nitrophenol. Sodium carbonate (0.25 M) was used to stop the reactions after the specified amount of time had passed. With a Rayleigh UV-2601 spectrophotometer (BFRL, Beijing, China), we determined the absorbance at a wavelength of 400 nm. The unit of enzyme activity was the quantity of p-nitrophenol emitted after 1 h of reaction for 1 mg of protein in 1 mL of sample [µMh × mg^−1^].

### 2.3. Concentration of Lactic Acid, SCFAs, and BCFAs

The following process was followed to prepare the samples for HPLC analysis: First, we vortexed a suspension of 0.5 g of sample in 3 mL of demineralized water (Vortex RS-VA 10, Phoenix Instrument, Garbsen, Germany). Following centrifugation (12,000 rpm, 20 min, Centrifuge MPW-251, MPW, Warsaw, Poland), the supernatant was filtered (0.22 m filters, ALWSCI Technologies, Shaoxing, China) and transported to sterile autosampler vials. The investigation used high-performance liquid chromatography (HPLC) using a Surveyor system (Thermo Scientific, Waltham, MA, USA). The technique employed the following equipment and conditions: an Aminex HPX-87H column (300 mm × 7.8 mm), a UV detector, 0.005 mL^−1^ sulfuric acid as eluent, a flow rate of 0.6 L min^−1^, and a duration of 40 min for analyzing a single sample.

### 2.4. Statistical Analysis

During the study, each measurement was conducted three times, and the data in the tables are reported as means ± SD. The Shapiro–Wilk test was used to assess the normality of the variable distribution, and Bartlett’s test was used to evaluate whether the variances were homogenous. After normality and equal variance were established, the data were examined using the one-way ANOVA test and Tukey’s post hoc test. Python 3.11 was used to conduct a statistical analysis with a significance level of *p* < 0.05.

## 3. Results

### 3.1. Determination of the Activity of Fecal Enzymes

The study set out to explore the intricate dynamics between a vegetable–fruit preparation enriched with RD and the activities of fecal enzymes in overweight children. By scrutinizing α-glucosidase, β-glucosidase, α-galactosidase, β-galactosidase, and β-glucuronidase, the research aimed to unravel the potential impact of this preparation on gut health.

The central hypothesis postulated that the consumption of the fortified preparation could induce discernible alterations in enzyme activities, favoring the reduction of potentially harmful enzymes while bolstering those beneficial to gut health. This hypothesis was corroborated through the meticulous analysis of enzyme activities pre- and post-intervention, coupled with comparisons between the RD group and the placebo group.

**Figure 1:**
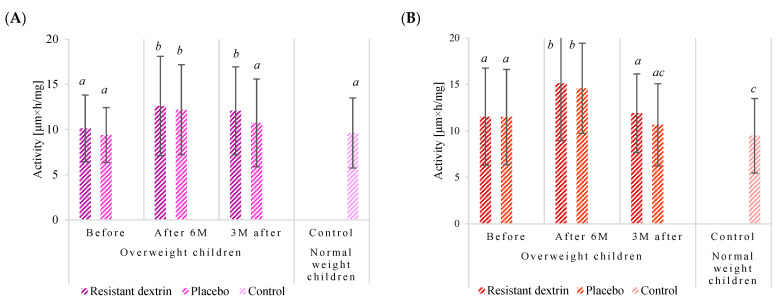

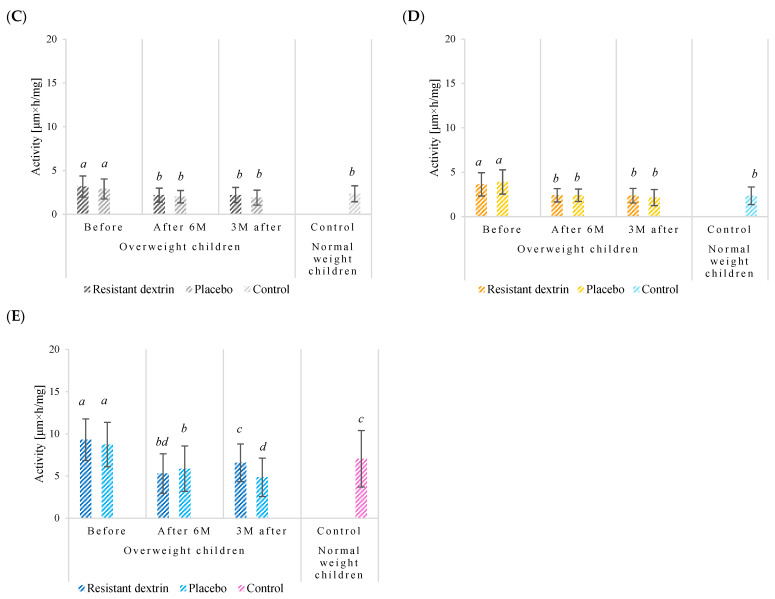
Activity of α-glucosidase (**A**), α-galactosidase (**B**), β-glucosidase (**C**), β-galactosidase (**D**), and β-glucuronidase (**E**) in fecal samples of overweight children receiving vegetable–fruit preparation with or without RD, and in fecal samples of children of normal weight. *^a^*^, *b*, *c*, *d*^: the results are statistically different from other results assigned with different letters (*p* < 0.05).

The results unveiled a complex interplay between the intervention and fecal enzyme activities (Figure 1A–E). Notably, in the RD group, α-glucosidase activity surged from 10.120 µMh/mg pre-intervention to 12.606 µMh/mg post-intervention, representing a substantial increase of approximately 24.56%. This surge was followed by a slight decline to 12.072 µMh/mg three months after the intervention ceased. Conversely, the placebo group displayed a similar pattern, albeit with a lesser magnitude of change, ascending from 9.387 µMh/mg to 12.188 µMh/mg post-intervention, then regressing to 10.728 µMh/mg three months later.

Intriguingly, while initial α-glucosidase activity was comparable between overweight and normal-weight children, post-intervention, overweight children exhibited higher activity, particularly in the RD group. This suggests that the intervention may have a differential impact on enzyme activities based on weight status.

Similarly, α-galactosidase activity mirrored the trends observed in α-glucosidase. In the RD group, α-galactosidase activity surged from 11.536 µMh/mg to 15.134 µMh/mg post-intervention, marking a significant increase of approximately 31.19%. Conversely, the placebo group demonstrated a parallel increase, albeit with a lesser magnitude. This trend was also observed in the subsequent decline post-intervention cessation.

The trajectory of β-glucosidase activity exhibited a contrasting pattern. Participants in the RD group experienced a notable decrease from 3.175 µMh/mg to 2.192 µMh/mg post-intervention, constituting a substantial decline of approximately 44.84%. Conversely, the placebo group displayed a comparable decrease in activity, albeit with a lesser magnitude. Notably, a higher proportion of participants in the RD group exhibited decreased β-glucosidase activity, suggesting a potential beneficial effect of RD.

The activity of β-galactosidase mirrored that of β-glucosidase, with participants in the RD group displaying a significant decrease from 3.642 µMh/mg to 2.410 µMh/mg post-intervention. Similarly, a higher proportion of participants in the RD group exhibited decreased activity, reinforcing the potential beneficial influence of RD.

Finally, β-glucuronidase activity followed a similar trajectory to the other enzymes, with a substantial decrease observed in the RD group post-intervention. Interestingly, overweight children exhibited higher baseline activity compared to their normal-weight counterparts, suggesting potential metabolic differences between the two groups.

Overall, while absolute values of fecal enzyme activities were lower in the RD group compared to the placebo group, the observed changes in enzyme activities were more favorable in the RD group. These findings suggest a potential beneficial effect of RD on the gut microbiota and metabolic health of overweight children.

**Figure 2:**
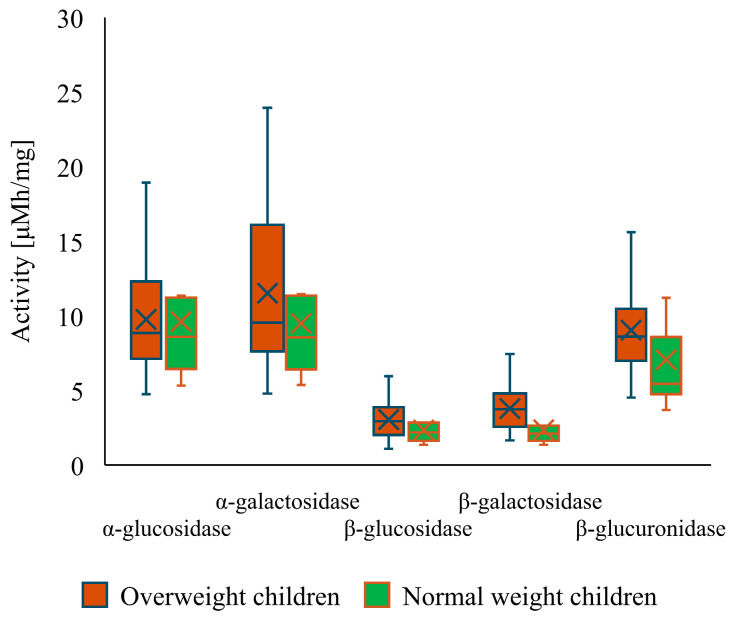
Activity of fecal enzymes in overweight versus normal-weight children.

**Figure 3:**
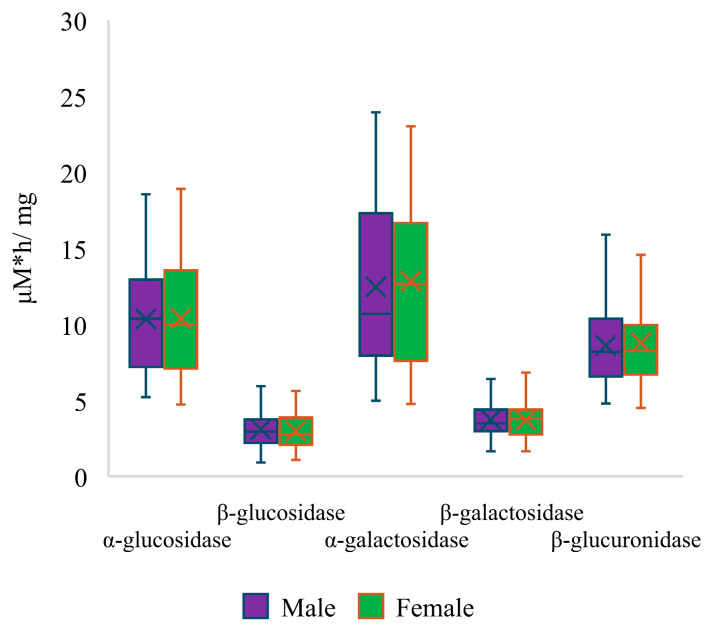
Comparison of activities of fecal enzymes differentiated between males and females.

Furthermore, a comparison between the activities of fecal enzymes of overweight (before intervention) and normal-weight children demonstrated that there are significant differences in the activities of certain enzymes (Figure 2). It was measured that the group of overweight children had significantly higher activities of highly mutagenic enzymes (β-glucosidase and β-glucuronidase). Moreover, the activities of α-galactosidase and β-galactosidase were also increased in said group. No difference was observed only for α-glucosidase. In addition, a comparison of enzymatic activity was carried out between male and female children (Figure 3), revealing no statistically significant differences between the two groups.

**Figure 4:**
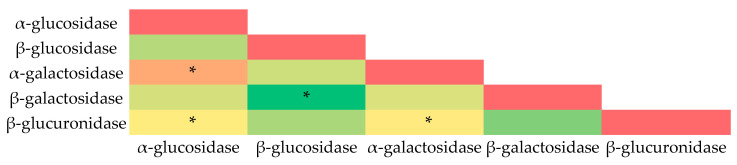
Pearson’s heatmap representing correlation between the activities of fecal enzymes. Color scale represents correlations found, where green colors indicate positive correlations (the stronger the shade, the stronger the correlation), yellow indicates neutral (no correlation), and red indicates negative correlation. *: *p* < 0.05.

A heat map was also created to determine possible trends and correlations between the tested fecal enzyme activities (Figure 4).

The strongest correlation was found between α-glucosidase and α-galactosidase activities. Surprisingly, there was a correlation between α-glucosidase, α-galactosidase, and β-glucuronidase activities (Figure 4). This may be due to the fact that the total bacterial count was higher in the normal-weight group, resulting in a higher number of β-glucuronidase-producing bacteria. However, α-glucosidase and α-galactosidase activities were significantly higher in the normal-weight group.

The observed results can be attributed to variations in BMI, dietary choices, and the balance between probiotic bacteria and less desirable species. These factors alter the levels and functions of many metabolites, promoting the presence of beneficial or possibly detrimental ones. Alterations in the microbiota are likewise linked to variations in pH levels within the colon, potentially impacting the functionality of certain enzymes. These findings corroborate the hypothesis that obesity has substantial effects on the synthesis of metabolites in the colonic region of children.

### 3.2. Determination of Concentrations of Lactic Acid, SCFAs, and BCFAs

The investigation delved into the intricate interplay between a vegetable–fruit preparation fortified with RD and the concentrations of lactic acid, SCFAs, and BCFAs in fecal samples obtained from overweight and obese children. A comprehensive analysis aimed to discern nuanced alterations in metabolic profiles induced by the intervention, with particular emphasis on individual-level effects within the RD and placebo groups, as well as a comparative assessment between overweight and normal-weight children.

### 3.3. Lactic Acid

**Figure 5:**
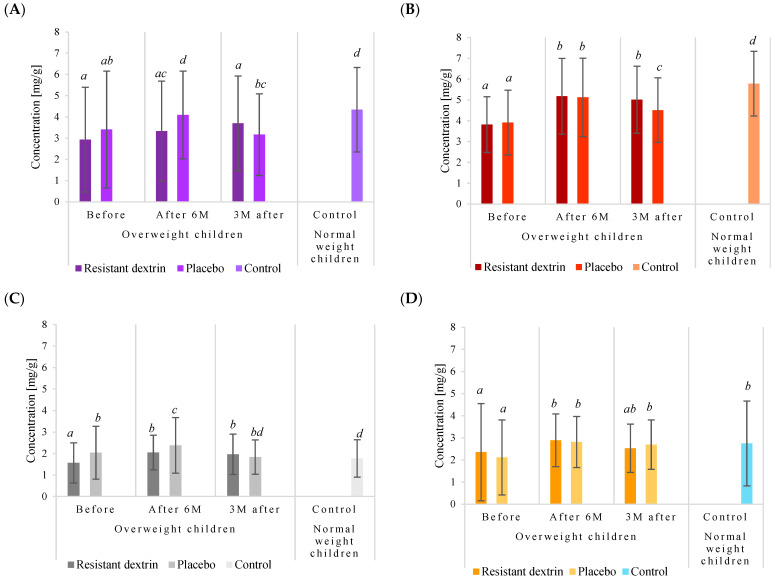

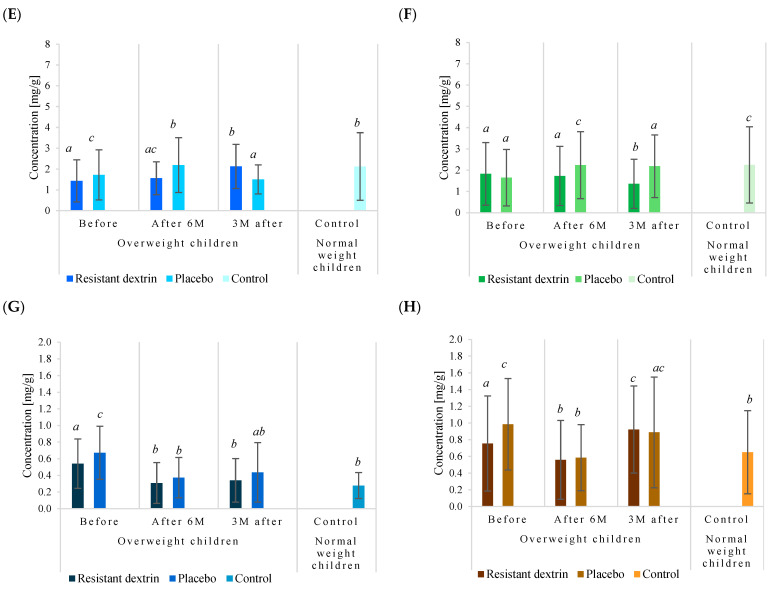
Concentration of lactic (**A**), acetic (**B**), formic (**C**), propionic (**D**), butyric (**E**), valeric (**F**), isovaleric (**G**), and isobutyric (**H**) acids in fecal samples of overweight children receiving vegetable–fruit preparation with or without RD, and in fecal samples of children of normal weight. *^a^*^, *b*, *c*, *d*^: the results are statistically different from other results assigned with different letters (*p* < 0.05).

Lactic acid, a key metabolite produced during carbohydrate fermentation, exhibited dynamic fluctuations in response to the dietary intervention (Figure 5A). In the RD group, lactic acid concentrations displayed a steady upward trajectory, escalating from a baseline of 2.931 mg/g before the intervention to 3.336 mg/g after 6 months, and further to 3.697 mg/g after 3 months post-intervention cessation. Conversely, the placebo group, while demonstrating slightly higher baseline concentrations, followed a similar pattern of increase post-intervention. Notably, lactic acid concentrations remained elevated compared to baseline even 3 months post-intervention cessation in the RD group, suggesting a sustained impact of the dietary intervention. Intriguingly, while overweight children initially exhibited lower lactic acid concentrations compared to their normal-weight counterparts, the RD group experienced a significant improvement over the study period, indicating a potential normalization of metabolic profiles.

### 3.4. Short-Chain Fatty Acids

Concentrations of SCFAs assessed during the intervention are displayed in Figure 5B–F. Acetic acid concentrations exhibited notable changes in response to dietary interventions. In the group receiving preparations with RD, acetic acid levels increased from 3.819 mg/g before intervention to 5.177 mg/g after 6 months, representing a substantial 35.55% rise (*p* < 0.05). However, a slight decrease of 3.26% was observed 3 months post-intervention. Similarly, the placebo group showed a comparable increase in acetic acid levels after 6 months of intervention, followed by a significant decrease to 4.508 mg/g 3 months post-intervention. These fluctuations underscore the dynamic nature of microbial fermentation and highlight the potential impact of RD on SCFA production.

Propionic acid concentrations also demonstrated significant changes throughout the intervention period. In the RD group, propionic acid levels increased from 1.567 mg/g to 2.048 mg/g after 6 months (*p* < 0.05), followed by a slight decrease of 3.92% post-intervention. Conversely, the placebo group exhibited a similar increase in propionic acid levels during the intervention, but a more substantial decrease to 1.837 mg/g 3 months post-intervention. These findings suggest differential effects of RD on propionic acid production compared to other dietary components.

Butyric acid concentrations exhibited distinct patterns of change in response to dietary interventions. While the RD group showed a non-significant increase in butyric acid levels during the intervention period, a significant surge was observed 3 months post-intervention, with levels reaching 2.128 mg/g (*p* < 0.05). In contrast, the placebo group demonstrated a significant increase during the intervention, followed by a decline below baseline levels post-intervention. These contrasting patterns underscore the complex interplay between dietary components, gut microbiota composition, and metabolic responses.

Formic acid concentrations increased significantly in both groups after 6 months of intervention, with a subsequent decrease post-intervention. However, the RD group exhibited a more substantial decrease compared to the placebo group, suggesting a potential role of RD in modulating formic acid production.

Valeric acid concentrations displayed differential responses to dietary interventions. While the RD group showed a decrease in valeric acid levels post-intervention, the placebo group exhibited a significant increase. These divergent responses underscore the need for further investigation into the specific mechanisms underlying the effects of RD on valeric acid metabolism.

### 3.5. Branched-Chain Fatty Acids

The concentrations of BCFAs, including isovaleric acid and isobutyric acid, underwent significant fluctuations post-intervention, shedding light on the intricate metabolic responses elicited by the dietary intervention (Figure 5G,H). Isovaleric acid concentrations exhibited a marked decrease after 6 months of intervention, followed by a subsequent increase 3 months post-intervention cessation, with a slight rise observed in the RD group. Similarly, isobutyric acid concentrations displayed a significant decrease after 6 months of intervention, followed by a notable increase 3 months post-intervention cessation. Nonetheless, no significant difference was found in the percentage of participants with decreased isobutyric acid concentrations between the RD and placebo groups, suggesting comparable metabolic responses to the dietary intervention.

**Figure 6:**
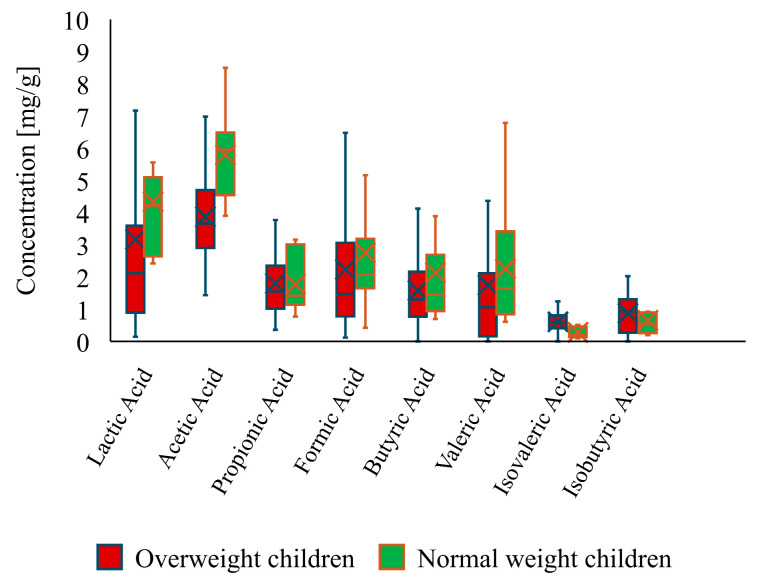
Concentrations of lactic acid, SCFAs, and BCFAs in overweight and normal-weight children.

Additionally, a comparison between the metabolic profiles of overweight and normal-weight children indicated that there are indeed significant differences in the concentrations of lactic acid, SCFAs (acetic, formic, butyric, and valeric acids) and BCFAs (isovaleric and isobutyric acids). The group of overweight children was characterized by lower concentrations of lactic acid (37.14% difference) and most other tested SCFAs (26.8–49.56% differences, with the exception of propionic acid), whereas the concentration of BCFAs was noticeably higher in said group, with a 33.49% difference for isobutyric acid and an over 100% difference for isovaleric acid (Figure 6).

**Figure 7:**
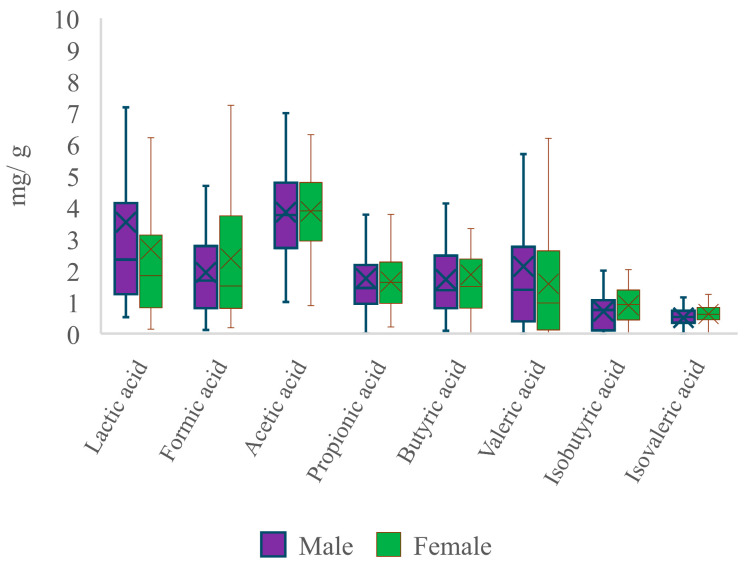
Concentrations of lactic acid, SCFAs, and BCFAs differentiated between males and females.

The study also assessed differences in fatty acid profiles between male and female groups to see if gender could have an impact (Figure 7).

**Figure 8:**
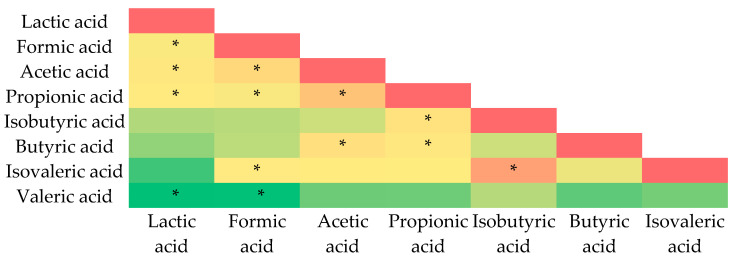
Pearson’s heatmap represents correlation between the concentrations of fatty acids. Color scale represents correlations found, where green colors indicate positive correlations (the stronger the shade, the stronger the correlation), yellow indicates neutral (no correlation), and red indicates negative correlation. *: *p* < 0.05.

The concentrations of lactic, formic, and isobutyric acids differed significantly between the research groups, as seen in Figure 7 (*p* < 0.05). Men exhibited a 24.3% greater concentration of lactic acid and a 35.1% higher concentration of valeric acid compared to females (*p* < 0.05). Conversely, the levels of formic, isobutyric, and isovaleric acids were 18.5%, 21.4%, and 19.1% lower in males, respectively (*p* < 0.05). The concentrations of acetic, propionic, and butyric acids did not show any significant differences across the groups. Various heat maps were then created to determine possible correlations between the fatty acid concentrations tested (Figure 8).

A significant association was seen between the SCFAs, as well as between the two measured BCFAs. The amounts of acetic and propionic acids showed the highest relationships, as calculated in Figure 8. Nevertheless, there was a significant correlation between lactic acid content and SCFAs, particularly formic, acetic, and propionic acids. There is an unambiguous relationship between the concentrations of isovaleric and isobutyric acids, which suggests that they are produced by the same bacteria that are linked to obesity.

The metabolic function of various metabolites, such as fatty acids, is influenced by the individual’s dietary choices, the composition of their gut microbiome, and the balance between beneficial probiotic bacteria and less favorable species. The findings corroborate the concept that obesity has substantial effects on the generation of metabolites in the colon among youngsters.

## 4. Discussion: Fecal Enzymes

Numerous factors, including diet, might affect how gut microbiota function. First, some microbes in the large intestine may become more enzymatically active as a result of dietary substrates [28,29]. Moreover, diet has the potential to modify the composition of gut microbiota by enhancing the prevalence of specific species and types of bacteria [30].

The large intestine bacteria’s enzymatic activity may lead to the creation of poisonous or possibly hazardous compounds for the human body [26]. The manufacture of carcinogens and other harmful chemicals often involves the enzymes of gut bacteria, which mostly belong to the classes of reductases and hydrolases. The enzymes β-glucuronidase (EC 3.2.1.31), β-glucosidase (EC 3.2.1.21), and β-galactosidase (EC 3.2.1.23) display the highest activity among them [23]. The elevated activity of these enzymes is frequently associated with specific alterations in the composition of the gut microflora, wherein less advantageous species start to prevail in the environment [27].

There are cases where the activity of certain enzymes increases. For example, individuals with colorectal cancer show a higher activity of β-glucuronidase and β-glucosidase caused by an increased number of *Clostridium* bacteria in the feces of patients, compared to bacteria isolated from healthy people [31,32]. This is supported by research that demonstrates that the *Clostridium* genus has the greatest activity of β-glucuronidase and β-glucosidase among the microbes of the large intestine. Additionally, it was proven that individuals with colorectal cancer had considerably more bacteria belonging to the genus *Clostridium* [33,34]. Such information is strongly consistent with the results of the previous paper, where the addition of RD decreased the number of *Clostridium* species and the activity of β-glucuronidase and β-glucosidase during an in vitro study [35], and later the administration of a vegetable–fruit preparation enriched with RD caused a significant decrease in the activity of both β-glucuronidase and β-glucosidase.

Fecal enzymes, such as β-glucosidase and β-glucuronidase, have the potential to be toxic to the host organism and may encourage the development of various cancers, as was previously described by [36]. β-glucosidase, like β-glucuronidase, is recognized for its ability to convert heterocyclic aromatic amines, polycyclic aromatic hydrocarbons, and some bile acids into carcinogenic substances, as well as produce aglycons. [36,37,38]. Additionally, it has been revealed that inhibiting β-glucosidase may both slow the development of cancer and make colorectal cancer more responsive to chemotherapy [39].

Modifying the composition of gut microbiota has an impact on the function of enzymes involved in breaking down carbohydrates. Therefore, supplementing with non-digestible carbohydrates like RD seems to be a feasible option for achieving this objective. [31,40].

Probiotic bacteria like *Lactobacillus* have been proven in multiple investigations [41,42,43] to reduce the activity of fecal enzymes. The findings disclosed that probiotic *Lactobacillus* (*Lacticaseibacillus casei*, *Lactiplantibacillus plantarum*, and *Lacticaseibacillus rhamnosus*) may significantly lower fecal β-glucuronidase activity in human subjects by over 50%, which is considered a significant anticarcinogenic effect [41]. It cannot be stated how the addition of RD influenced the balance of gut microbiota of children, as this was not part of the current paper, but it can be suspected based on the previous in vitro study on the subject that the abundance of the *Lactobacillus* genus may be increased in fecal samples of children supplemented with the preparation enriched with RD [35]. Thus, such an explanation can be used to justify a decrease in the activity of β-glucuronidase.

Similar to this, research conducted by [42] showed that supplementing with *Lactiplantibacillus plantarum* and prebiotic (acacia gum) together dramatically reduced the enzymatic functions of procarcinogenic β-glucosidase and β-glucuronidase to a greater degree than either probiotic or prebiotic supplementation individually. Since the results of the presented paper are comparable, it can be stated (with caution, however) that RD may have the ability to increase the number of certain *Lactobacillus* species and act as prebiotic, and finally to reduce the activities of β-glucosidase and β-glucuronidase, which was observed during the research.

Furthermore, there are studies that indirectly corroborate the advantages of probiotic strains derived from the *Bifibacterium* and *Lactobacillus* genera [43,44]. Three different strains of bacteria—*Levilactobacillus brevis* (isolated from kimchi), *Bifidobacterium bifidum*, and *Bifidobacterium longum* (isolated from newborn feces)—were found to lack β-glucuronidase activity in these studies. These results imply that the procarcinogenic glucuronidases may decrease, as observed in the presented paper, once the gastrointestinal tract has an increased abundance of the genera *Bifibacterium* and *Lactobacillus*.

Contrary to the results presented in this paper, in a study by [45] it was observed that NUTRIOSE^®^ (Roquette Frères, Lestrem, France; RD) caused the increased concentration of α-glucosidase and β-glucosidase in healthy men. Although the increased concentration of α-glucosidase may be interpreted as beneficial as it may help to ferment RD and induce the production of lactic acid and SCFAs, the increased concentration of β-glucosidase is potentially harmful due to its involvement in the production of procarcinogenic compounds. It can be debated if such an effect was caused by the different type of RD used (wheat origin), or if some other factors were involved during the study, as the RD from potato starch (used in the presented study) promoted a reduction in the activity of β-glucosidase.

In contrast, the study by [46] found that a diet rich in resistant starch administered to healthy individuals caused a significant decrease in bacterial β-glucosidase, which was consistent with the results obtained for the group of children receiving the preparation with RD that was presented in this paper. Thus, it can be suggested that the RD from potato starch has similar effects on β-glucosidase as a resistant starch.

Interestingly, a study conducted on elderly patients who received inulin showed different results [47], with no effect on β-glucosidase and β-glucuronidase whatsoever. It can be suspected that the treatment of their patients was too short (only 19 days) as compared to 6 months of administration of RD during the presented study; however, changes in the microbiota composition were already noticed. Another factor may be that the microbiota in the elderly react slightly worse to certain treatments, and a higher dose of prebiotic should be administered to achieve the desired effects. Nonetheless, such diverse results for different prebiotic substances suggest that there are other possible factors involved in the regulation of enzymatic activity in feces.

Worth mentioning is the fact that there are a variety of different sources of starch that may be used in the production of RD. According to a number of studies, RD that is derived from wheat or maize starch similarly has prebiotic qualities. Ref. [48] conducted a randomized, placebo-controlled trial on healthy patients, which disclosed that NUTRIOSE^®^ (sugar-free RD from what or maize starch) significantly increased the numbers of beneficial bacteria (*Lactobacillus* and *Bifidobacterium*), while simultaneously reducing the counts of *Clostridium perfringens*. In addition, the effect on the enzymes found in the feces was proven by a rise in the activity of the α-glucosidase enzyme following the use of NUTRIOSE^®^.

It is furthermore significant to note that the percentage of participants with increased enzyme activities does not necessarily indicate the magnitude of the increase or the clinical significance of the change. Moreover, the results for α-glucosidase and β-galactosidase were higher in the placebo group, which suggests that other factors may be influencing the activities of these enzymes. In summary, the results of the study suggest that the vegetable–fruit preparation enriched with RD may have a positive effect on the activities of some fecal enzymes in overweight children, particularly for β-glucosidase and β-glucuronidase, and can potentially be a viable dietary strategy to modulate the activity of fecal enzymes in overweight children. Nonetheless, the effects were still relatively similar in both the intervention and placebo groups, which suggests that the vegetable–fruit preparation alone was having an effect on the activities of fecal enzymes. Thus, further research is needed to determine the clinical significance of these changes and to investigate the potential health benefits of the observed effects.

Regarding the comparison of the activities of fecal enzymes in overweight and normal-weight children, the data on the subject are limited. Even though changes in the intestinal microbiota have been considered important topics in recent years, some branches are still lacking quality research. Most studies focus either on the abundance of certain microorganisms, or on the concentration of lactic acid, SCFAs, or BCFAs. It can be stated that the comparison of the activity of fecal enzymes between overweight and normal-weight children was one of the first experiments in that regard. Nonetheless, it makes speculation about results challenging, given how diverse the results of other studies concerning fecal enzymes are. It can be hypothesized that the activity of fecal enzymes such as α-glucosidase in normal-weight children should be high, especially if the diet is rich in fibrous compounds, which stimulate the growth of bacteria that ferment fiber and produce SCFAs as a byproduct. Similarly, it is logical that the activity of potentially mutagenic enzymes such as β-glucosidase and β-glucuronidase was lower in normal-weight children, as genera such as *Clostridium* should be less abundant in their gastrointestinal tract.

Furthermore, it can be the case that the consumption of a higher amount of food and being overweight may lead to a higher abundance of total bacteria in the intestines, which increases the overall activity of fecal enzymes. In that case, given that dysbiosis is often associated with overweight status, less desirable bacteria can dominate the environment, thus increasing the activity of potentially mutagenic enzymes (β-glucosidase and β-glucuronidase).

## 5. Discussion: Lactic Acid, SCFAs, and BCFAs

The primary bacterial products of the digestion of proteins and carbohydrates in the gut are SCFAs, which significantly contribute to host–microbiota interactions. Contrarily, BCFAs, primarily isovaleric and isobutyric acids, are generated in lower quantities, and little is known about their fecal levels in various human populations, gut-bacteria-generating populations, or effects on health [49].

It is well known that intestinal microbiota ferment branched-chain amino acids (valine, leucine, and isoleucine) to create BCFAs. According to the scientific literature, the levels of branched-chain amino acids (BCFAs) rise from the proximal colon to the distal colon and feces in the human gut, where BCFA fermentation is mostly carried out by the genera *Bacteroides* and *Clostridium* [50]. When protein-fermenting bacteria were cultivated in vitro with peptides as their primary carbon source at a pH of 6.8, BCFA generation was exhibited; however, the introduction of starch at a lower pH of 5.5 inhibited BCFA creation in these cultures [51].

According to evidence from certain dietary interventions conducted on both human subjects and animals [52,53], diets high in protein and low in complex carbohydrates, like the western diet, result in higher concentrations of BCFAs. In general, dietary supplementation with complex carbohydrates such as RD that can pass through the colon causes a decrease in fecal levels of BCFAs, while dietary supplementation with protein tends to increase the production of these substances by intestinal microbiota [54,55]. In addition, even though they could be performing crucial roles in the gut environment and might perhaps serve as indicators of the microbial metabolism occurring in the gut, BCFAs have received significantly less attention than major SCFAs (acetate, propionate, and butyrate).

Furthermore, BCFAs have been proposed as markers of colonic protein fermentation, a process that results in the concurrent production of other protein fermentation products such as p-cresol, ammonia, phenol, or biogenic amines, molecules that can potentially harm intestinal flora by causing cell damage and even promote the development of cancer [56,57]. High levels of isovalerate in feces have been linked with human depression and altered cortisol levels [58], and more recently, it has been suggested that BCFAs may be crucial in the regulation of glucose and lipid metabolism, which can contribute to improved insulin sensitivity in individuals with disturbed metabolism [59].

There are, however, no comprehensive data on fecal levels of BCFAs throughout life or in various health statuses, despite our understanding of their biosynthesis routes. Therefore, a significant amount of research should be conducted to unveil all the interactions involving BCFAs.

As hypothesized in the previous section, effects of the RD added to the vegetable–fruit preparation on metabolites of fecal microbiota in overweight children were observed. However, the scope of the RD influence varied significantly between tested acids and phases of the study, which was described in detail in the previous section. Nonetheless, it can be stated that in general, the addition of RD to the vegetable–fruit preparation had a minor positive effect, and moreover did not cause any adverse effects, which is a significant part of any investigation, especially on human subjects.

Furthermore, the administration of a pure vegetable–fruit preparation without any additives caused a similar effect to the preparation with RD, even though the effects were not as long-lasting as those in the RD group, which were detectible after 3 months from the last administration of the preparation. Nonetheless, it is a matter of great interest given that such an effect may be helpful in, e.g., stabilizing microbiota after antibiotic treatments or adverse shifts in the balance of gut microbiota.

Another human trial by [60] aimed to investigate the effects of RD (and other prebiotics) on immune function and intestinal microbiota structure in perioperative patients with colorectal cancer (CRC). The supplementation in the preoperative period was associated with an increased genus-level abundance of *Bifidobacterium and Enterococcus* and reduced genus-level abundance of *Bacteroides*. Prebiotic intervention did not significantly alter the abundance of *Enterococcus* in the postoperative period, whereas the non-prebiotic group had a decline in *Bacteroides* and an increase in *Enterococcus.* As *Bifidobacterium*, *Enterococcus*, and *Bacteroides* are genera that produce high amounts of lactic acid and SCFAs, it can be concluded that with the increase in the abundance of bacteria, the concentrations of certain acids would further be increased, which would confirm the results presented in this paper.

Similar effects were also seen in a more recent study by [61], which investigated how RD alters the metabolism and composition of gut bacteria. In total, 20 healthy volunteers were administered RD (14 g/d NUTRIOSE^®^) for 4 weeks, which was comparable to doses administered in the study described in this article (15 g/d). Similarly, the results were evaluated before, during, and after administration. The RD caused alterations in microbial metabolism and composition, including an uptick in the number of species capable of generating short-chain fatty acids (including *Bifidobacterium longum*, *Eubacterium eligens*, *Roseburia* spp., and *Ruminococcus* spp.). Although bacteria species in the tested fecal samples were not evaluated as a part of this paper, it can be suspected that they were also affected (i.e., based on the results of the previous in vitro study [35]), and had an effect on the concentration of lactic acid and SCFAs, which was measured.

Studies on the effect of RD are limited; however, it is possible to draw certain conclusions based on the comparison of the effects of the tested RD with other known prebiotic compounds such as inulin or resistant starch. For example, the results presented in a study by [53] are evidently comparable with the results presented in this article. The study [53] associated the consumption of resistant starch with increased levels of acetate and butyrate, which was likewise the case for the tested RD. Moreover, [53] observed a decrease in concentrations of BCFAs (isobutyrate and isovalerate), which is strongly consistent with the results presented in this paper. Similar results, especially in the case of butyrate, were also obtained by [62], who also examined the effects of resistant starch on the metabolism of gut microbiota.

Furthermore, a clinical trial conducted by [63] disclosed that inulin caused similar changes in the concentration of SCFAs, especially acetate and butyrate, which was similarly achieved by the addition of RD to vegetable–fruit preparations in the study described in this paper. Thus, it can be concluded that tested RD has comparable effects to known prebiotics such as resistant starch.

Moreover, in further research, there are several matters that should be considered when it comes to studying the metabolites of microbiota, which may reduce the high standard deviation during the whole course of study. The most important factor is possibly controlling the diets of the participants of the study, which is especially essential during long investigations such as the one presented in this paper. Dietary advice may not be enough to maintain a healthy and balanced diet, especially when children are the group of focus. Moreover, it is easier to assess the effects of a given dietary additive, such as a vegetable–fruit preparation, if a strict diet is administered to every participant. Otherwise, the proportion of macro-elements in the diet cannot be determined, which may influence the results, and thus cause a high variation of results within the tested group. Furthermore, during the year, the availability of different foodstuffs also varies significantly, so during the winter months, fruits and vegetables are not only less available but also more expensive, which may reduce the frequency of their occurrence in everyday diet. Contrarywise, during the warmer part of the year, the availability of plant-based products, fruits, and vegetables increases, which may increase the frequency of their administration to children and thus cause additional alterations in gut microbiota. Notably, the changes in microbiota during winter may be relatively negative, while during spring and summer these seasonal diet changes can significantly increase the amount of fiber in the child’s diet, which further influences the balance of gut microbiota and may generate false-positive results in the study, increase the variation in the studied group, and disturb the proper conclusions of the study. Seasonal changes in the availability of certain foodstuffs may be even stricter in other parts of Europe, or other parts of the world.

Comparable conclusions were drawn in a study by [64], where the stability of the human fecal microbiome was thoroughly examined. It was discovered that although the microbiome has stable elements (i.e., prevalent and stable species of bacteria), there are many variables that are associated with less resistant bacteria that can fluctuate over time and change according to environmental conditions such as seasonal changes in diet, which was the case for the study conducted in the presented paper.

Furthermore, a recent study by [65] confirmed the abovementioned results. By long-term interval analysis, it was observed that the microbiota of individuals change, and moreover, these changes are increasing over time. Given the 1-month time intervals used in the study, it can be stated that similar changes could easily occur in the cohort of children participating in the presented study, since its entirety lasted 9 months for each individual. Therefore, in future research evaluating health markers that are strongly dependent on gut microbiota, the diet should be a basic factor that is controlled throughout the study, because otherwise there are imminent changes in the microbiome of individuals that may have impact on the results.

Another important factor, which is usually omitted in most studies, is the standardization of fecal samples, which was also not fully performed in the presented article. Although the samples were cleaned from various undigested parts of food, the water content of the samples was not examined or standardized. Therefore, some samples displayed an apparently higher content of water, whereas others were relatively solid. Nonetheless, to obtain more reliable and less varied results, fecal samples should be dried and milled to obtain a set of samples with a similar texture and water content, which would decrease the chance of errors. The concentrations of fatty acids in fecal samples are relatively low, which renders them especially prone to being diluted, and therefore not being detected.

The correlation between stool consistency and the composition of fecal microbiota was demonstrated in a study by [66], where it was concluded that indeed, the richness of the gut microbiome is linked with the consistency of the stool. As mentioned above, this may also be the cause of the relatively large standard deviation of the results of the fatty acids investigation, as the consistency of fecal samples varied significantly.

Moreover, similar correlations were found in a study by [67], who found that fecal SCFA concentrations are correlated with fecal water content, which further confirms the importance of the standardization of fecal samples in future studies.

Furthermore, this phenomenon was confirmed for other compounds such as bile acids. In a study by [68], it was observed that different results of concentrations of bile acids that were measured in the same samples varied only by standardization method according to dry weight or wet weight. Given that the samples described in this paper were not standardized, it increases suspicion that the results obtained from their analysis yielded a wide range of different results and thus, a relatively high standard deviation.

Furthermore, the storage methods of the stool samples can also influence their microbial community. In a study by [69], it was investigated how different methods of stool handling and storage may influence the bacterial community within samples. The proposed method that reduced the variability of microbiome data was freezing the samples within 15 min from defecation and storing them in a frost-free freezer for less than 3 days. During the studies included in the presented article, such a method was not possible to implement, given that the samples had to go through different laboratories and be transported at least twice, not to mention their long storage time in a freezer. According to the study by [69], such handling of samples may increase the variability of the microbiome, and thus influence the tested metabolites present in the fecal samples, which again may be a cause of a relatively wide range of results within the tested group of children. To further investigate the effects of the addition of RD to vegetable–fruit preparations, the assessment of the activity of selected fecal enzymes was conducted.

In conclusion, the increased concentrations of lactic acid and most of the SCFAs that were observed during the presented study suggest that beneficial changes in gut microbiota transpired. As stated before, increased concentrations of abovementioned metabolites have several health-promoting, anticarcinogenic, and immunogenic effects, and moreover can possibly regulate food intake by increasing satiety in children by increasing the levels of peptide YY (satiety hormone) [70,71]. Furthermore, the decreased concentrations of BCFAs obtained in the presented study may be of interest, since as mentioned before they possess potentially procarcinogenic effects linked with the degradation of proteins in the gut and the accumulation of several harmful byproducts [72]. Nonetheless, since the concentration of BCFAs is not standardized, it w hard to assess if the reduction that occurs has a positive or negative impact on health, since some studies also highlight their positive roles in human metabolism [49].

On the other hand, when metabolites in overweight and normal-weight subjects are compared, a certain amount of caution should be considered. As of yet, there are no standards for the typical concentration of fatty acids in the gastrointestinal tract or stool samples. On one hand, it can be suspected that lean individuals should have a healthier microbiota composition, yielding a higher concentration of SCFAs or lactic acid, simply because of the higher viability of bacteria that produce said acids (*Bifidobacterium*, *Lactobacillus*, and *Enterococcus*). Such a hypothesis would be consistent with the results presented in this paper, where normal-weight children had higher concentrations of lactic acid and SCFAs, whereas their concentrations of BCFAs were lower than in overweight children. However, it can also be hypothesized that being overweight and consuming more macronutrients may cause higher numbers of bacteria in the large intestine, and even though the ratio of beneficial genera is lower, their total amount could still be higher in comparison to normal-weight people, thus increasing the production of metabolites. Nonetheless, studies on that topic are often not comprehensible as they focus on specific phyla or genera and omit the holistic image of the gut microbiome [73,74]. It is also highlighted by researchers that other factors such as transit time or intestinal absorption may play a vital role in varying levels of SCFAs in the colon [75,76].

Such a case was highlighted by the results in the study by [76], who observed a pattern where obese individuals had higher concentrations of fecal SCFAs when compared to lean subjects. Moreover, it was strongly highlighted that such differences were possibly not directly correlated with diet or different SCFA absorptions, but with the fact that overweight and obese individuals produce more fecal SCFAs due to differences in compositions of microbiota.

There is, however, a limited amount of data when it comes to children under the age of 10, which were the participants of the study presented in this article. It may be the case that at such a young age, there are different patterns or mechanisms that determine whether normal-weight children should produce more or less SCFAs than overweight children. Furthermore, even though the number of participants of the presented study was high, the number of children in the control group was much lower, which could be a cause of certain error. It may be the case that the metabolism of children is somewhat different, and being overweight does not directly correlate with a higher amount of fatty acids present in the large intestine due to their faster absorption rate, or that their gut microbiota are less active, thus producing less metabolites.

Considering the results and their constraints, it is important to highlight the need for more research that compares the metabolite profiles formed by the microbiota of the digestive tract in individuals with varying anthropometric status. This may contribute to a better insight into the area of dependencies in the human gastrointestinal environment.

## 6. Conclusions

In summary, there is a significant disparity in the metabolites detected in the fecal samples of children with obesity compared to those with a healthy BMI. Children who are obese showed significantly elevated levels of branched-chain fatty acids (BCFAs), which are linked to an imbalance in gut bacteria. The findings demonstrated that the concentrations of lactic acid and SCFAs in these children are significantly lower when compared to those of children with a healthy weight. The administration of a vegetable–fruit preparation enriched with RD from potato starch increased the concentrations of lactic acid and SCFAs (except valeric acid), while decreasing concentrations of BCFAs in overweight children, and positively impacted the longevity of the results obtained during its administration (at least 3 months after administration).The vegetable–fruit preparation without RD from potato starch had a similar impact on the tested health markers as the preparation with RD, but its effects on overweight children were not maintained 3 months after administration. The concentrations of lactic acid and SCFAs were higher in the normal-weight children when compared to overweight children, whereas the concentrations of BCFAs were lower in the group of normal-weight children.

In addition, the levels of advantageous α-glucosidase and α-galactosidase activities were significantly elevated in the cohort of children with a healthy weight, while they were reduced in the obese children. By contrast, the levels of β-glucuronidase activity, identical to β-glucosidase, were shown to be higher in the population of children with a high BMI. These findings provide additional evidence supporting the adverse consequences associated with the high occurrence of obesity, which may result in the emergence of serious medical conditions. Furthermore, the addition of RD from potato starch to a vegetable–fruit preparation had a positive effect on the activities of fecal enzymes in overweight children and caused prolonged effects of increased α-glucosidase and α-galactosidase activities (3 months after the last ingestion). Moreover, the activities of fecal enzymes were significantly higher in the cohort of overweight children compared to the group of normal-weight children (especially β-glucosidase, β-galactosidase, and β-glucuronidase).

Overall, it can be concluded that the addition of RD from potato starch to a vegetable–fruit preparation may be a viable strategy to improve selected health markers of overweight children, and thus possibly influence the process of losing weight.

Nevertheless, additional research is required to gain a deeper understanding of the complex network of interactions among the microbiota of the digestive tract, their environment, and the physical characteristics of humans.

## Figures and Tables

**Table 1:** Summary characterization of the participants of the study *.

	Overweight and Obese Children		Normal Weight Children	
Gender	Male	Female	Male	Female
	46	51	5	6
Age [years]	8.67 ± 1.05	8.26 ± 1.33	7.58 ± 1.23	7.48 ± 0.92
Weight [kg]	51.55 ± 11.16	44.53 ± 10.76	24.7 ± 3.38	24. 3 ± 2.96
Height [m]	1.43 ± 0.10	1.37 ± 0.10	1.26 ± 0.07	1.26 ± 0.06
BMI [kg × m^−2^]	25.06 ± 3.67	23.49 ± 3.45	15.5 ± 0.72	15.15 ± 0.71

* The table consists of average values with standard deviation (SD). kg: kilogram; m: meter, BMI: Body Mass Index.

**Table 2:** Dietary information about vegetable–fruit preparations *.

Nutritional Information per 100 g	Apple–Peach–Parsnip	Apple–Carrot–Chaenomeles	Apple–Cherry–Carrot-Banana
Energy	283 kJ/67 kcal	262 kJ/62 kcal	278 kJ/66 kcal
Fat	<0.5 g	<0.5 g	<0.5 g
of which saturated fats	<0.1 g	<0.1 g	<0.1 g
Carbohydrates	14 g	13 g	14 g
of which sugars	8 g	8.5 g	9.2 g
Fiber	2.8 g	2.5 g	2.1 g
Protein	0.7 g	0.5 g	0.6 g
Salt	<0.01 g	0.05 g	0.03 g
Composition	Apple puree, peach juice from concentrate, parsnip, dextrin from potato starch, juices from concentrate from apple and lemon, and vitamin C	Purees from apple, carrot, and Chaenomeles, apple juice from concentrate, dextrin from potato starch, and vitamin C	Purees from apple, sour cherry, carrot, and banana, dextrin from potato starch, apple juice from concentrate, and natural sour cherry flavoring
Volume	85 mL		

* Vegetable–fruit preparations were prepared for the study by TYMBARK-MWS, Poland. g: gram; kJ: kilojoules; kcal: kilocalories.

## Data Availability

The data analyzed are publicly available in source articles and data citations were included in the reference list. The data that support the findings of this study is also available from the corresponding author, K.Ś. and M.W.
